# Intraocular pressure and axial length changes during altitude acclimatization from Beijing to Lhasa

**DOI:** 10.1371/journal.pone.0228267

**Published:** 2020-01-29

**Authors:** Yuan Wu, Ci Ren Qiong Da, Jiang Liu, Xiaoming Yan

**Affiliations:** 1 Department of Ophthalmology, Peking University First Hospital, Beijing, China; 2 Department of Ophthalmology, Tibet Autonomous Region People’s Hospital, Lhasa, Tibet Autonomous Region, China; Bascom Palmer Eye Institute, UNITED STATES

## Abstract

**Purpose:**

To investigate changes in intraocular pressure (IOP) and axial length (AL) on the ascent to high altitude from Beijing to Lhasa.

**Patients and methods:**

Twenty volunteers (17 men, 3 women) who had been sent to work in Lhasa, Tibet for more than 6 months were enrolled. One of their journeys from Beijing to Lhasa was chosen as the time for the examination. IOP, AL, corneal curvature (K), and blood pressure (BP) were measured in Beijing (altitude 43 m) and Lhasa (altitude 3658 m). Their first examination was conducted at least 1 day before arriving in Lhasa and the second examination after they had stayed in Lhasa for 7 days. The data from the highland and lowland examinations were analyzed with a paired-sample T test and Pearson’s correlation coefficient was calculated for the association between IOP and average BP.

**Results:**

The mean IOP was 12.65±2.34 mmHg in Beijing and 14.85±3.1 mmHg in Lhasa. The mean AL was 24.61±1.50mm in Beijing, and 24.98±1.45 mm in Lhasa. The IOP and AL showed a significant elevation in highland compared with lowland (*P*<0.05). The mean K was 43.58±2.25 D in Beijing and 43.56±2.21 D in Lhasa and no significant difference was found in this study (P>0.05). A positive correlation between variance of IOP and ACD was found (r = 0.475, *P*<0.05) and no correlation between IOP and average BP was noted.

**Conclusions:**

High altitude may lead to a small but significant change in IOP and axial length. However, the shape of the corneal surface was not influenced by the hypobaric and hypoxic conditions.

## Introduction

High altitude has a systemic effect on human beings, including the visual system. But the relationship between IOP and altitude has been inconsistent. Multiple studies have shown varying effects of IOP at high altitudes [1][2][3][4]. Furthermore, researchers have found that IOP was reduced within hours of ascent, and recovered during acclimatization[5][6].

China is a vast country, with varying geographic characteristics. Many cities on the Qinghai-Tibetan plateau, such as Lhasa and Shigatse, have an elevation that is over 3600 meters. Every year, many tourists and workers travel between the eastern lowlands and the western highlands of China. A high altitude has diverse systemic and ophthalmic effects on individuals. Ophthalmic effects include changes in IOP[5][7], the tear film[8], cornea[7], choroid[9] and retina[10][11][12]. Government staff members that were sent to the highlands from the lowlands, have complained about blurred vision in Lhasa. It has been suggested that refractive system could also be affected by high altitudes. Until now, there has been no consistent result on the effect of altitude on IOP and no documentation about eye shape changes.

Lhasa, with an altitude of 3658 m and nearly 1 million population, is one of the highest metropolises in the world. In this study, healthy volunteers from Beijing, a lowland with an elevation of 45 m in eastern China, were enrolled in this study. The IOP and axial length of eye balls were measured in both Beijing and Lhasa, and changes were documented and compared. The aim of this study is to objectively assess human responses to hypoxic and hypobaric stresses.

## Materials and methods

20 volunteers from Beijing who had worked in Lhasa for > 6 months were enrolled between March to July 2017. All subjects were adult Han Chinese, who had a normal slit lamp examination, a best-corrected visual acuity (BCVA) of >20/40 and an IOP < 21 mmHg. Subjects with a history of intraocular surgery and any ocular conditions other than refractive errors were excluded. One of their journeys from Beijing to Lhasa by flight was chosen as the time for the examination. Ophthalmic examination of IOP, biometric parameters, corneal curvature (K), central corneal thickness (CCT) and blood pressure (BP) were measured in Beijing (3 pm-4 pm) 1 day before they arrived in Lhasa. The IOP, biometric parameters, K and blood pressure were measured again in Lhasa (3 pm-4 pm) after the volunteers had stayed in Lhasa for approximately 7 days. Beijing and Lhasa are in the same time zone making the issue of jet lag mute. All measurements were measured at the same time in each city. Both eyes underwent the same ophthalmic examinations and only right eye data was selected for analysis. Values of IOP were measured three times using two identical applanation tonometers (AT900, Haag-Streit, Koniz, Switzerland), and K was measured three times using two identical autorefractometers (KR-8900, Topcon, Tokyo, Japan) in Beijing and Lhasa. Biometric parameters, including the AL, anterior chamber depth (ACD), lens thickness (LT), and vitreous chamber depth (VCD) of the eye balls, were measured with an A-mode ultrasonic pachymeter in Beijing (SW-2100, Souer, Tianjin, China) and an A-mode ultrasonic pachymeter (Compact Touch; Quantel Medical, Bozeman, MT, USA) in Lhasa. The mean of at least 10 readings were used for each eye. CCT was measured with a noncontact pachymeter (NT-530P, Nidek, Aichi, Japan) in Beijing only due to limited equipment available in Lhasa. The average BP (ABP) was calculated from diastolic blood pressure (DBP) and systolic blood pressure (SBP) using the formula: average BP = DBP +1/3(SBP-DBP). All examinations were repeated three times in both cities. The means of the measurements were used as the ocular parameters for analysis. Before the study, a consistency check between the two examiners were done at Beijing, and the results were highly coherent.

This study was approved by the Medical Ethics Committee of the Tibet Autonomous Region People’s Hospital. All participants gave informed written consent according to the tenets of the Declaration of Helsinki.

### Statistics

SPSS (ver. 14.0; SPSS Inc., Chicago, IL, USA) was used for all statistical analyses. Data exhibiting a normal distribution are expressed as the mean±standard deviation. Comparisons were performed between the two measurement using paired Student’s t-test. A value of P<0.05 was considered statistically significant. Pearson’s correlation coefficient was calculated to determine the association between IOP, AL, ACD and average BP.

## Results

More than 40 volunteers registered for this study but many subjects were excluded due to incomplete data collected during the trips between Beijing and Lhasa. Only 20 volunteers completed the data collection process, including 17 men and 3 women. The mean age was 42.56±5.5 years. The average IOP of the volunteers in Beijing was 12.65±2.34 mmHg and increased to 14.85±3.1 mmHg in Lhasa (Table 1), with a significant difference between the two data groups. Elongations of the axial length and ACD were found between Beijing and Lhasa, but differences were not found for corneal curvature, lens thickness and VCD (Table 1).

**Table 1:** IOP and ocular parameters examined in Beijing and Lhasa.

	IOP (mmHg)	Corneal curvature (D)	Axial length (mm)	Length of anterior chamber (mm)	Thickness of lens (mm)	Length of vitreous chamber (mm)
**Beijing**	12.65±2.34	43.58±2.25	24.61±1.50	2.80±0.46	4.23±0.29	17.37±1.41
**Lhasa**	14.85±3.1	43.56±2.21	24.98±1.45	3.39±0.37	4.04±0.32	17.36±1.37
**T value**	-4.104	0.298	-3.823	-6.824	-0.395	0.146
***P* value**	0.001	0.769	0.001	0.000	0.698	0.886

**Fig 1:**
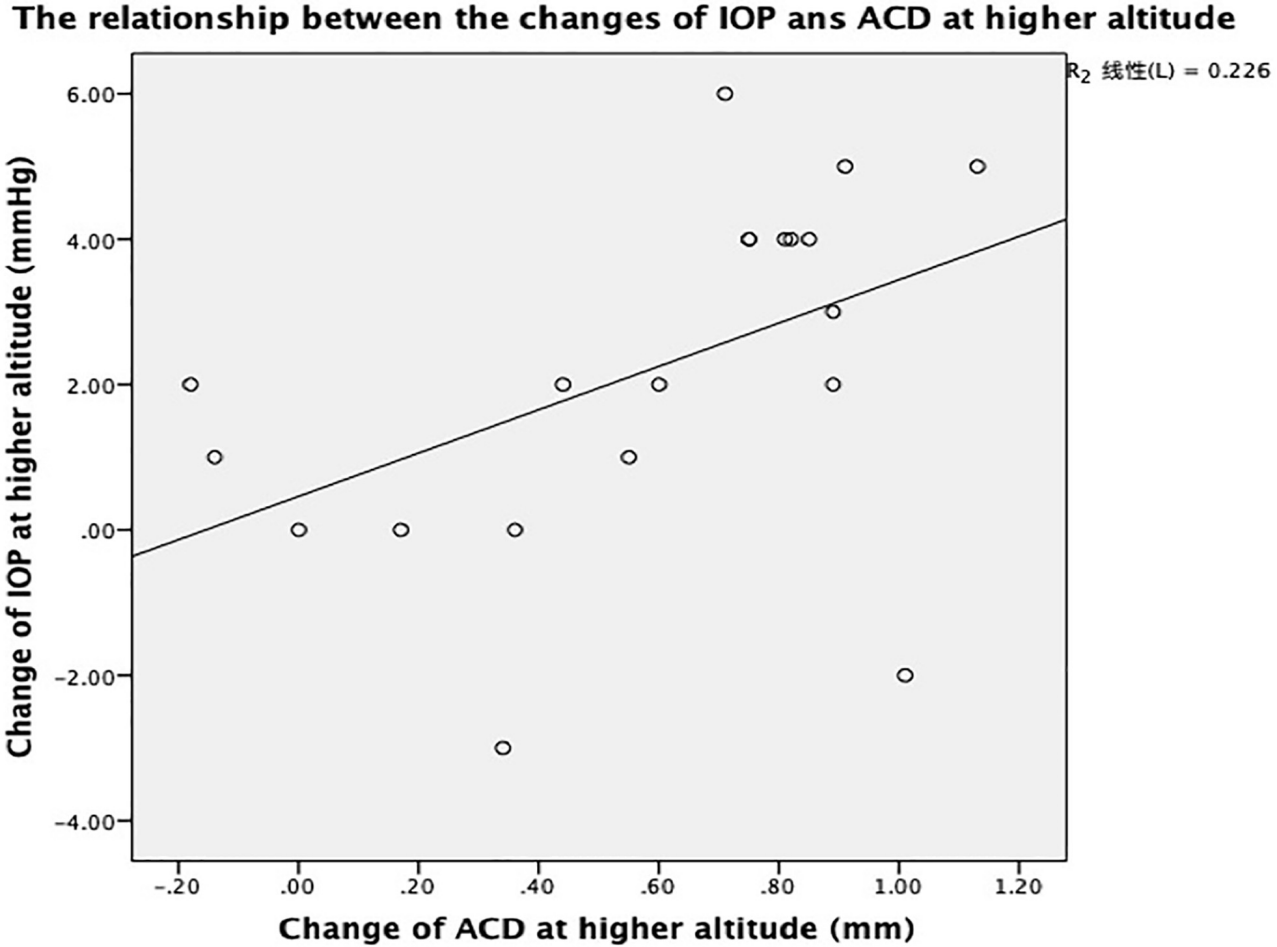
Plot showing change of IOP versus change of ACD.

The ABP was 91.4±9.7 mmHg in Beijing and 105.3±15.9 mmHg in Lhasa. There was no relationship between the variance of IOP and the variance of axial length and the variance of the ABP, except for the variance of ACD (Table 2). The correlations between variance of IOP and ACD were positive (Fig 1).

**Table 2:** The correlation between the variance of IOP and related factors.

	Variance of AL	Variance of ACD	Variance of ABP	Corneal thickness
**Variance of IOP**	r = 0.316	r = 0.475	r = -0.121	r = 0.302
	P = 0.175	P = 0.034	P = 0.613	P = 0.195

## Discussion

The likely cause of the changes in IOP with variations in altitude is fluctuations in barometric pressure. Three conditions have been used to examine this issue before, flight environment, artificial hypobaric chamber and ascending altitude (Table 3). The most ideal research condition for this is during air flight. Bayer et al. measured the changes in IOP in 25 healthy volunteers during a routine flight. They found that IOP decreased significantly after a two-hour flight, and that this decrease in IOP was sustained at a significant level after landing, where the altitude was the same as the place at which they got onto the plane[13]. The fact that IOPs showed a difference despite being in the same altitude, showed that there are multiple factors, such as physical activity and sympathetic stimulation during travel, which might contribute to the changes in IOP together. Another ideal research model is an artificial hypobaric environment. Karzdag et al. evaluated the IOP changes of 26 young male participants in a chamber simulating hypoxic conditions (exposed to a pressure equivalent to 9144m). They found that the IOP was significantly greater than before and after exposure[14]. The average increase in IOP was 2.44 mmHg, and this was similar to the results from our study.

**Table 3:** Results about IOP change as a function of altitude in different conditions.

Research	number of subjects (n)	subjects	environment	method of IOP measurement	time	IOP
**Bayer A**[13]	20	volunteers	3048 m during flight	Tono-Pen XL	1 h	no change
					2 h	-13.4%
					landing	-15.8%
**Karzdag R**[14]	26	volunteers	9000m mimic chamber	Tono-Pen XL	1–3 min	14.9%
**Somner JE**[1]	104	climbers	5200 m climbing	Tono-Pen XL	1–4 days	7.9%
**Bosch MM**[12]	25	climbers	5300 m climbing	Tono-Pen XL	many days	9.7%
**Willlmann G**[20]	14	climbers	4559 m climbing	Goldmann	1–3 days	no change
**Karakucuk S**[2]	40	climbers	2800m (from 1080m)	Tono-Pen XL	1 day	no change

The most common research model was conducted on climbers. Some studies[5][6] showed that IOP increased significantly from baseline after acute exposure to a higher altitude, and they showed a decrease after acclimation. Others have shown no change in IOP when climbing. However, these conclusions may have been affected by the environmental conditions associated with high altitudes. Cold environments could result in a drop in IOP due to a fall in episcleral venous pressure[15]. Exercise and fatigue could also cause a decrease in IOP[16]. IOP could also be affected by hypoxia and mountain sickness through vascular mechanisms. A number of studies chose lowlanders who had acute high-altitude exposure as their subjects, which could be complicated with another interference factor such as the altered autoregulation of blood flow in the eye. Different degrees of such factors may lead to different results and may neutralize the increase in IOP, which may be the reason why previous studies have been inconclusive. Until now, the net effects of altitude on IOP has been unknown.

In this study, healthy volunteers who lived in the highland city for 6 months were selected. It is presumed that these subjects have high hematocrit levels and other physiological characteristics adjusted to hypobaric and hypoxic conditions. All ophthalmic examinations were performed in indoor clinics. We expect our data is more credible since the subjects were merely affected by altitude.

In this study, we found that immigrating from lower land to higher land may cause an increase in IOP, and the variance of IOP was related with the variance in ACD. There may be two reasons to explain the IOP changes.

First, increased IOP may be caused by increased systemic blood pressure. Some studies have shown that a 10 mmHg increase in SBP and DBP was associated with 0.2 and 0.4 mmHg increase in IOP, respectively[17]. In this study, all the subjects had an elevated ABP of 15 mmHg. High blood pressure may result in changes in aqueous humor formation by increased capillary pressure in the ciliary body and episcleral venous pressure, which is important in regulating the outflow of the aqueous. The intraocular aqueous may be the reason of increasing IOP and ACD. At the same time, hypoxia may affect aqueous humor dynamics by increasing episcleral venous pressure. Hypoxia will narrow the vessels by decreasing pO2 and increasing pCO2[18].

Second, the change in CCT may also affect IOP measures. Some research has shown that the CCT increased after exposure to high altitudes[7][19][20]. It is known that IOP can be overestimated when the CCT is increased and underestimated when it is decreased. Karadag et al. found that even after correcting for CCT, IOP still increased in a simulated high altitude environment[14]. Unfortunately, we could not test the CCT of subjects in Lhasa due to a lack of the required instruments. However, a CCT related change in IOP may be at play in high altitudes.

Many studies have used a Tono-Pen, which is a handheld electronic instrument with an advanced ergonomic design, to measure IOP in different environments[13]. This device is compact and portable and able to be used outside, but the value of a Tonopen is not the same as that of Goldmann applanation tonometry (GAT). Some research has shown that the Tonopen may overestimate the IOP value compared to GAT[21]. The measurement of GAT was based on Imbert-fick principle. The area of flattening is constant and a variable force based on Newtonian force is measured. Thus, it is regarded as the standard of measurement for IOP for almost 60 years[22]. In 2017, Willmann et al. might’ve been the first research group to use GAT to evaluate IOP changes after high altitude exposure[4]. The current study might be the second to evaluate IOP related to altitude and the results could have greater validity.

Another concern about the accommodation of altitude is the change in K and axial length. In this study, we did not find statistically significant changes in K, but we did find changes in AL, which have not been reported to date. It is hard to compare the changes in these ocular parameters at different altitudes because of device limitations. The autofractometer and ultrasonic pachymeters are difficult to carry with mountain climbers. In this study, we chose two ophthalmology clinics in the two cities to implement such examinations. We found that the axial length and anterior chamber length had significant elongation under high-altitude conditions. The elongation of axial length corresponded to an increased diopter of -0.5 to -1D, which could be the reason of blurred vision in such subjects.

This transformation of eye shape may be due to a quick adaptation of the eyeball under a lower barometric pressure. The physical characteristics of the eyeball are similar to a latex balloon filled with water. The size of the eyeball would have enlarged a little to maintain a lower pressure because the barometric pressure of Lhasa is only 60% of Beijing. Secretion and drainage of aqueous fluid may achieve a new balance and the decreased pressure difference between the inside and outside of the eyeball would be greater in Lhasa than Beijing. At the same time, we also note that the difference in measurement instruments and examiners may contribute to the prolongation of axial length at Lhasa. One of the examiners might touch the cornea more lightly because of his operating habits.

### Study limitations

One of the shortcomings of this study is having different examiners at the two examination clinics and this could contribute to examiner bias. Before starting the research, we performed a consistency check between the two examiners. One group of 10 subjects had their IOP tested by the two examiners, and there was no significant difference between the measurements obtained by the two examiners. The difference in IOP measurements between the examiners can be assumed to be very small during this research. Furthermore, the instruments used for measuring keratometry and axial lengths were different between the two clinics. The difference in axial length measurements could be confounded by the different measurement techniques applied by the different examiners. In a similar study, two identical devices were used at two different places, and this was also acceptable considering travel limitations [9]. Some equipment could not travel with the subjects from Lhasa to Beijing, and we admit that this was a systematic error in this study. We hope more portable devices can be designed and applied in studies in the future.

## Conclusions

In this study, we concluded that a change in altitude resulted in a statistically significant change in IOP and axial length, which could be due to ocular adaption to hypobaric conditions and may be the reason for blurred sight. We could not identify the underlying mechanism for this phenomenon; however, high blood pressure and elevated episcleral venous pressure may play an important role. Future research with an increased sample size and decreased measurement bias is needed to further validate this.

## Supporting information

S1 Table The raw data of this research. (XLSX)
