# Correction: Intraocular pressure and axial length changes during altitude acclimatization from Beijing to Lhasa

**DOI:** 10.1371/journal.pone.0231208

**Published:** 2020-03-26

**Authors:** Yuan Wu, Ci Ren Qiong Da, Jiang Liu, Xiaoming Yan

## Notice of republication

An incorrect version of S1 Table was published in error. This article was republished on March 11, 2020, to correct for this error. Please download this article again to view the correct version.
